# Efficient Expression and Processing of Ebola Virus Glycoprotein Induces Morphological Changes in BmN Cells but Cannot Rescue Deficiency of Bombyx Mori Nucleopolyhedrovirus GP64

**DOI:** 10.3390/v11111067

**Published:** 2019-11-15

**Authors:** Jinshan Huang, Na Liu, Fanbo Xu, Ellen Ayepa, Charles Amanze, Luping Sun, Yaqin Shen, Miao Yang, Shuwen Yang, Xingjia Shen, Bifang Hao

**Affiliations:** 1 Jiangsu Key Laboratory of Sericultural Biology and Biotechnology, School of Biotechnology, Jiangsu University of Science and Technology, Zhenjiang 212018, China; 13793161442@163.com (N.L.); shenxjsri@163.com (X.S.); bfhao@just.edu.cn (B.H.); 2 Key Laboratory of Genetic Improvement of Sericulture in the Ministry of Agriculture, Sericultural Research Institute, Chinese Academy of Agricultural Science, Zhenjiang 212018, China

**Keywords:** Ebola virus, glycoprotein, baculovirus, signal peptide

## Abstract

Ebola virus (EBOV) disease outbreaks have resulted in many fatalities, yet no licensed vaccines are available to prevent infection. Recombinant glycoprotein (GP) production may contribute to finding a cure for Ebola virus disease, which is the key candidate protein for vaccine preparation. To explore GP_1,2_ expression in BmN cells, EBOV-GP_1,2_ with its native signal peptide or the GP64 signal peptide was cloned and transferred into a normal or gp64 null Bombyx mori nucleopolyhedrovirus (BmNPV) bacmid via transposition. The infectivity of the recombinant bacmids was investigated after transfection, expression and localization of EBOV-GP were investigated, and cell morphological changes were analyzed by TEM. The GP64 signal peptide, but not the GP_1,2_ native signal peptide, caused GP_1,2_ localization to the cell membrane, and the differentially localized GP_1,2_ proteins were cleaved into GP_1_ and GP_2_ fragments in BmN cells. GP_1,2_ expression resulted in dramatic morphological changes in BmN cells in the early stage of infection. However, GP_1,2_ expression did not rescue GP64 deficiency in BmNPV infection. This study provides a better understanding of GP expression and processing in BmN cells, which may lay a foundation for EBOV-GP expression using the BmNPV baculovirus expression system.

## 1. Introduction

Ebola virus (EBOV), a member of the family *Filoviridae*, is the causative agent of Ebola hemorrhagic fever, a disease that causes significant morbidity and mortality in humans and nonhuman primates, with a human fatality rate as high as 90%, including the 2014 outbreak in West Africa with more than 27,000 cases and 11,000 deaths [1]. Currently, there are no licensed vaccines for EBOV, and our understanding of viral pathogenesis remains limited. The EBOV genome includes seven genes, 3′-NP-VP35-VP40-GP-VP30-VP24-L-5′ [2], and the GP gene encodes three GP precursors. Processing of GP in cells leads to the production of various forms as the protein is transported through the endoplasmic reticulum (ER) and Golgi apparatus to the plasma membrane [3]. The full-length transmembrane GP, which mediates virus entry, including attachment, entry, and fusion, is the only glycoprotein on the surface of the Ebola viron [4]; thus, EBOV GP is the key candidate protein for vaccine preparation in mammalian or plant cells [5,6,7].

An N-glycosylated precursor form of GP (GP-pre), which is found in the ER, is further processed to the fully glycosylated, uncleaved form in the Golgi apparatus (GP_0_). Similar to all other Class I viral fusion proteins, GP_0_ is then cleaved by furin-like proteases to generate GP_1_ (130 kDa), the role of which appears to involve receptor binding [2], and transmembrane GP_2_ (24 kDa). These two subunits are linked through intersubunit disulfide bonds and noncovalent interactions in the Golgi complex [8]. It is suggested that EBOV GP_1,2_ mediates initial attachment to host cells via lectin binding [9,10,11], and these initial attachment events are followed by internalization of the virus via macropinocytosis and trafficking of virus-containing macropinosomes toward the endolysosomal pathway [12,13,14]. Within the acidified endosome, GP_1_ is digested by cysteine proteases cathepsins B and L, which cleave off the mucin-like domain as well as other regions, producing a 20-kDa core and exposing the putative receptor-binding domain [15,16], which interacts with Niemann-Pick C1 protein to mediate EBOV entry into endolysosomes [15].

Bombyx mori nucleopolyhedrovirus (BmNPV) is a critical viral pathogen for silkworms, and the BmNPV expression system has been applied for expressing many foreign proteins, as either a cell culture system or in an insect larval system, in East Asia. Comparing to Autographa californica multiple nucleopolyhedrovirus (AcMNPV) expression system, the advantage of BmNPV expression system is that the silkworm can be raised easily in the room on a large scale with a lower cost than that of cell culture. In our previous research, we observed BmNPV entry into host cells via macropinocytosis [17]. It is known that mature macropinosomes fuse with lysosomes after macropinocytosis [18], and BmNPV GP64 requires a low pH of 4.5, similar value to that of the lysosome [18], for conformational change [19], suggesting that GP64 mediates viron membrane fusion with the lysosomal membrane. Thus, one key question is whether EBOV-GP can be expressed and processed efficiently in BmN cells and whether GP can substitute for the function of GP64 in BmNPV infection.

In this report, full-length EBOV-GP_1,2_ was expressed using the BmNPV Bac-to-Bac expression system, in which GP_1,2_ was expressed with its native signal peptide or with the GP64 signal peptide. The results indicated that the viral signal peptide of GP64 leads to efficient GP_1,2_ expression on the cell membrane; malfunction of the native signal peptide was confirmed because the distribution of the full-length protein in the cytoplasm was uniform. Nonetheless, the localization did not affect expression and cleavage of GP_1,2_. GP_1,2_ expression induced dramatic morphological changes in BmN cells, but it was unable to rescue GP64 deficiency in BmNPV infection. These results contribute to our understanding of EBOV-GP and may promote its application.

## 2. Materials and Methods

### 2.1. Cell Line and Viruses

BmN cells [17] (stored in our lab) were cultured in TC-100 (AppliChem, Darmstadt, Germany) insect medium supplemented with 10% FBS (Gibco BRL, Gaithersburg, MD, USA) at 27 °C using standard techniques. BmNPV bacmid-BmBacJS13 was constructed previously [20]. The Ebola virus glycoprotein gene (*gp*_1,2_) is derived from EBOV Zaire strain (GenBank accession no. KM655246.1) with an adenine insertion. In EBOV replication, an additional A residue is inserted into the coding sequence during transcription of the GP gene by the viral polymerase, to express the full-length GP_1,2_ in BmN cells, an adenine was inserted in the sequence.

### 2.2. Gp64-Knockout Bacmid Construction

A *gp64*-deletion bacmid was constructed using the chloramphenicol resistance gene (*CmR*) to replace *gp64*, as described [21]. Specifically, the upstream arm and downstream arm primer pair were designed to amplify a linear fragment containing *CmR* and with 50 bp of upstream and 50 bp of downstream flanking sequences of *gp64* (Table 1, nucleotides homologous to the *gp64* region are in italic font). Using these primers, a 1-kb PCR product containing the *CmR* gene was amplified from pBeloBac11 (Thermo Fisher Scientific, Waltham, MA, USA). The linear PCR product was gel-purified and transformed into *Escherichia coli* BW25113 containing the BmBacJS13 bacmid and the helper plasmid pKD46, which provides the phage λ Red recombinase [21]. *gp64*-deletion bacmids were obtained by homologous recombination in *E. coli* and screened using kanamycin and chloramphenicol resistance. Recombinant bacmids were identified by PCR with a GP64UP primer and CmRP primer, and the correct bacmid was designated BmBac^∆gp64^.

### 2.3. Generation of Recombinant Bacmids

To generate a GP_1,2_ expression recombinant BmNPV bacmid, the full-length *gp*_1,2_ gene with its native signal peptide (EBOV-SP_gp1,2_-*gp_1,2_*) or the BmNPV gp64 signal peptide (EBOV-SP_gp64_-*gp_1,2_*) was amplified and cloned into donor plasmid pFBD-egfp, where the *egfp* gene is downstream of the P10 promoter of pFastBacDUAL (Thermo Fisher Scientific, MA, USA). The resulting plasmids were subsequently electroporated into *E. coli* DH10B harboring Bm-Bacmid and helper plasmids to generate recombinant bacmids.

Briefly, the gp64 promoter containing its own signal peptide was amplified using a primer pair (Table 1, PGP64-F and PGP64-SPR) and cloned into pFBD-egfp using *Stu*I and *Xba*I sites to generate plasmid pFBD-egfp-SP_gp64_. *gp1,2* without the native signal peptide was amplified using primers GP1,2-noSPF and GP1,2-R (Table 1) and inserted into pFBD-egfp-SP_gp64_ using *Xba*I and *Hin*dIII sites to generate the transfer vector pFBD-egfp-SP_gp64_-gp_1,2_. The gp64 promoter was amplified with primers PGP64-F and PGP64-R (Table 1) and cloned into pFBD-egfp using *Stu*I and *Xba*I sites; full-length *SP_gp1,2_-gp_1,2_*was amplified using primers GP1,2-F and GP1,2-R (Table 1) and subsequently cloned into the vector to generate pFBD-egfp-SP_gp1,2_-gp_1,2_. pFBD-egfp-SP_gp64_-gp_1,2_ and pFBD-egfp-SP_gp1,2_-gp_1,2_ were transformed into DH10Bac competent cells containing the BmNPV bacmid-BmBac^∆gp64^ or BmBacJS13 and helper plasmid. The recombinant bacmids BmBac^∆gp64^-egfp-SP_gp64_-gp_1,2_, BmBac^∆gp64^-egfp-SP_gp1,2_-gp_1,2_, BmBac-egfp-SP_gp64_-gp_1,2_ and BmBac-egfp-SP_gp1,2_-gp_1,2_ were identified by PCR. As a control, BmBac-egfp was generated by transposition of pFBD-egfp.

### 2.4. Transfection and Infection of BmN Cells

BmN cells (3 × 10^5^ cells per 35 mm well) were transfected with 2.0 µg of the appropriate bacmid DNA (BmBac^∆gp64^-egfp-SP_gp64_-gp_1,2_, BmBac^∆gp64^-egfp-SP_gp1,2_-gp_1,2_, BmBac-egfp-SP_gp64_-gp_1,2_, and BmBac-egfp-SP_gp1,2_-gp_1,2_, and BmBac-egfp) using 6 µL of Lipofectamine (Thermo Fisher Scientific, MA, USA) and incubated for 4 h. The transfection mixtures were removed, and 2 mL of fresh medium containing penicillin/streptomycin at a final concentration of 50 units/mL and 50 µg/mL, respectively, were added. The virus-containing supernatant was collected from transfected BmN cells at 120 h post-transfection (p.t.) and used to infect cells in a 75-cm^2^ flask for virus amplification. BVs were collected at 96 h p.i., and titers were determined by the end-point dilution method.

### 2.5. GP_1,2_ Expression Analysis in BmN Cells

BmN cells (10^5^ cells per well) were infected with BmBac-egfp-SP_gp64_-gp_1,2_ and BmBac-egfp-SP_gp1,2_-gp_1,2_ or the control virus BmBac-egfp at a multiplicity of infection (MOI) of 5. The infected cells were harvested and centrifuged at 4000× *g* for 10 min at different time points p.i. The pelleted cells were lysed with 100 µL of PBS (pH 7.4), and 5 µL of the sample was analyzed by reducing gel, nonreducing gel, or native gel. Additionally, 5 µL of the samples were mixed with 1 µL PNGase F (Sigma-Aldrich, St. Louis, MO, USA) and incubated for 4 h at 37 °C, then the samples were subjected to SDS-PAGE gel. After separation, the proteins were transferred onto a PVDF membrane via semidry electrophoresis transfer for western blot analysis. A monoclonal anti-GP (Ebola/Zaire/1976) antibody (Cambridge Bio, Suzhou, China) and polyclonal rabbit anti-GP (Ebola/Zaire/1976) antibody (Sino Biological, Beijing, China) were used as the primary antibody, and an alkaline phosphatase-conjugated goat anti-mouse antibody or anti-rabbit antibody (SABC, Shanghai, China) was used as the secondary antibody. An anti-BmNPV VP39 antibody was used as the control. The EBOV-GP_1,2_ signal was detected with a nitro blue tetrazolium/5-bromo-4-chloro-3-indolyl β-D-galactopyranoside kit (SABC, Shanghai, China).

### 2.6. Immunofluorescence Analysis of GP_1,2_ Localization

BmN cells (4 × 10^4^) were seeded in 35-mm confocal dishes (Nest biotechnology, Changzhou, China), cultured overnight and infected with BVs of BmBac-egfp-SP_gp64_-gp_1,2_ and BmBac-egfp-SP_gp1,2_-gp_1,2_ or BmBac-egfp for 1 h. Unattached viruses were removed, and the cells were washed twice with TC100 insect medium without FBS. The cells were then incubated in TC100 insect medium with 10% FBS at 27 °C and prepared for confocal imaging at 48 h p.i. Briefly, the cells were fixed with 4% paraformaldehyde solution for 15 min, and then were permeabilized with 0.1% Triton X-100 for 30 min. After washing with phosphate-buffered saline (PBS, pH 7.4), the cells were labeled with the monoclonal anti-GP antibody (1:500 dilution) for 2 h and washed twice; an Alexa Fluor 555 labeled-secondary antibody in PBS containing 0.2% BSA was added for 1 h at room temperature. Nuclei were stained with 1 µg mL^−1^ Hoechst (Sigma-Aldrich, Saint Louis, MO, USA), and images were captured under a confocal laser scanning microscope (Leica SP8).

### 2.7. Electron Microscope Analysis

BmN cells (1 × 10^6^) were infected with BmBac-egfp-SP_gp64_-gp_1,2_, BmBac-egfp-SP_gp1,2_-gp_1,2_, or control virus BmBac-egfp at an MOI of 5 and harvested at 24 h p.i. and 72 h p.i. The samples were processed for transmission electron microscopy (TEM) examination.

## 3. Results

### 3.1. Construction of the gp64-Null Bacmid BmBac^∆gp64^ and Bacmids Harboring the Ebola GP_1,2_ Gene

**Figure 1:**
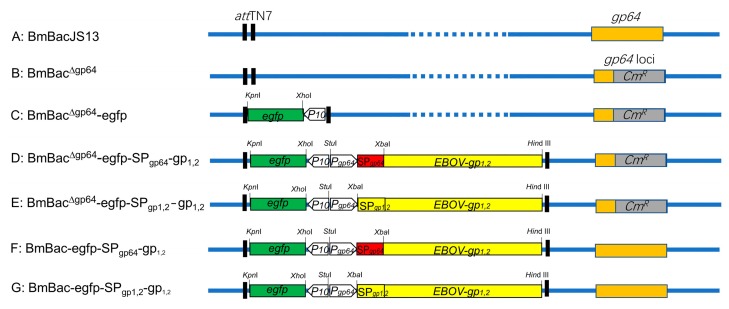
Schematic diagram of recombinant bacmid construction. The *gp64* gene (khaki boxes) in wild type bacmid (**A**) was replaced by *chloramphenicol resistance gene (CmR)* (**B**, grey boxes). Ebola virus (EBOV)-gp_1,2_ (yellow boxes) linked to the GP64 signal peptide (red box) or the native signal peptide was driven by the gp64 promoter and placed between the indicated restriction sites, and were transposed into BmBacJS13 (**A**) or BmBac^∆gp64^ (**B**) to generate recombinant bacmids BmBac^∆gp64^-egfp-SP_gp64_-gp_1,2_ (**D**), BmBac^∆gp64^-egfp-SP_gp1,2_-gp_1,2_ (**E**), BmBac-egfp-SP_gp64_-gp_1,2_ (**F**), BmBac-egfp-SP_gp1,2_-gp_1,2_ (**G**), egfp was transposed into gp64 null bacmid to generate BmBac^∆gp64^-egfp (**C**). The genomes of the BmNPV bacmids are represented by blue lines.

To ascertain whether GP_1,2_ can rescue the deficiency of GP64-null BmNPV in BmN cells, the recombinant bacmid BmBac^∆gp64^ was constructed, with *gp64* replaced by *CmR* (Figure 1A,B). The recombinant bacmid was examined by PCR and processed for transposition. The 1932-bp Ebola *gp_1,2_* gene without a signal peptide was amplified and cloned into the pFBD-egfp donor plasmid along with the gp64 promoter and the GP64 signal peptide (Figure 1D,F). Similarly, the 2031-bp full-length *gp_1,2_* gene containing its native signal peptide was amplified and cloned into the pFBD-egfp donor plasmid along with the gp64 promoter (Figure 1E,G). Subsequently, the Ebola gp1,2 expression cassettes were introduced into BmBacJS13 via transposition. The resulting bacmids, BmBac-egfp, BmBac^∆gp64^-egfp-SP_gp64_-gp_1,2_, BmBac^∆gp64^-egfp-SP_gp1,2_-gp_1,2_, BmBac-egfp-SP_gp64_-gp_1,2_, and BmBac-egfp- SP_gp1,2_-gp_1,2_ (Figure 1C–G), were identified by PCR.

### 3.2. EBOV-GP is Expressed and Cleaved in BmN Cells but Cannot Rescue GP64 Deficiency

**Figure 2:**
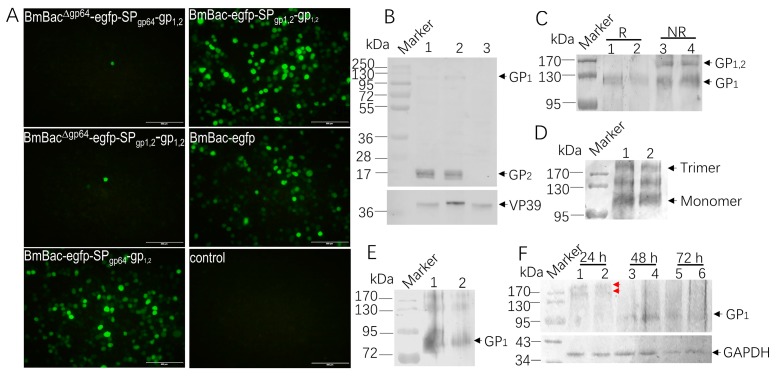
Expression of glycoprotein (GP) in BmN cells. (**A**) Fluorescence of transfection with different constructs in BmN cells. Fluorescence was recorded at 120 h p.t.; bar, 500 µm. (**B**) GP_1,2_ expression in infected BmN cells. BmN cells were infected with BmBac-egfp-SP_gp64_-gp_1,2_ (lane 1) and BmBac-egfp-SP_gp1,2_-gp_1,2_ (lane 2) or BmBac-egfp (lane 3) and harvested at 48 h p.i.; the cells were lysed and subjected to SDS-PAGE and western blot analysis with a monoclonal antibody against GP and BmNPV VP39. (**C**) Mobility of the GP_1,2_ protein in the SDS-PAGE gel presence or absence of the reducing reagent dithiothreitol (DTT). BmN cells were infected with BmBac-egfp-SP_gp64_-gp_1,2_ (lane 1, lane 3) and BmBac-egfp-SP_gp1,2_-gp_1,2_ (lane 2, lane 4) and harvested at 48 h p.i., then cell lysis were mixed with DTT (R) or without DTT (NR) protein sample buffers, heated at 95 °C for 5 min, and then subjected to SDS-PAGE followed by western blot analysis using a polyclonal antibody against the Ebola GP protein. (**D**) Analysis of trimerization of GP in BmN cell (48 h p.i.). Western blot analyses of GP proteins separated by native gel followed by western blot analysis using a polyclonal antibody against the Ebola GP protein. Lane 1, BmBac-egfp-SP_gp1,2_-gp_1,2_, lane 2, BmBac-egfp-SP_gp1,2_-gp_1,2_. (**E**) Deglycosylation assay on GP in BmN cells. 5 µL lysed cells samples (48 h p.i.) were mixed with 1 µL PNGase F and incubated for 4 h at 37 °C, then the samples were subjected to SDS-PAGE gel and western blot using a polyclonal antibody against the Ebola GP protein. Lane 1, BmBac-egfp-SP_gp1,2_-gp_1,2_, lane 2, BmBac-egfp-SP_gp1,2_-gp_1,2_. (**F**) Expression profiles of GP in BmN cells. BmN cells were infected with BmBac-egfp-SP_gp1,2_-gp_1,2_ (lane 1, lane 3, and lane 5) and BmBac-egfp-SP_gp1,2_-gp_1,2_ (lane 2, lane 4, and lane 6), and harvested at indicated time point, samples were mixed with protein sample buffers with DTT and heated at 95 °C for 5 min, and then subjected to SDS-PAGE followed by western blot analysis using a polyclonal antibody against the Ebola GP protein.

To investigate the function of GP_1,2_ in BmNPV infection, the recombinant bacmids were transfected into BmN cells. Although no amplification was observed for BmBac^∆gp64^-egfp-SP_gp64_-gp_1,2_ and BmBac^∆gp64^-egfp-SP_gp1,2_-gp_1,2_ at 120 h p.i. (Figure 2A), massive fluorescence was produced after transfection of BmBac-egfp-SP_gp64_-gp_1,2_, BmBac-egfp-SP_gp1,2_-gp_1,2_, and BmBac-egfp, while no fluorescence was observed in the uninfected cells (Figure 2A, control). These results suggest that the function of GP64 may not be rescued by EBOV-GP_1,2_. To further analyze EBOV-GP_1,2_ expression in BmN cells, BmBac-egfp-SP_gp64_-gp_1,2_, BmBac-egfp-SP_gp1,2_-gp_1,2_, and BmBac-egfp were infected into cells, which were collected at 48 h p.i. and examined by SDS-PAGE and western blot of protein extracts. The anti-BmNPV VP39 antibody showed efficient infection of BmN cells by BmNPV, and two bands were detected using the monoclonal anti-GP antibody. A band below 130 kDa and a small band located between 28 and 17 kDa were detected, with approximate molecular weights of 128 kDa (GP_1_) and 20 kDa (GP_2_) (Figure 2B). However, these specific bands were not detected in the control sample BmBac-egfp, indicating that GP_1,2_ was expressed in BmN cells and cleaved into GP_1_ and GP_2_.

To further verify whether GP proteins in the BmN cells are cleaved, we compared the mobility of GP protein in BmN cells preparations in the presence or absence of the reducing reagent dithiothreitol (DTT). For the weak reaction of monoclonal antibody to GP_1_, polyclonal rabbit anti-GP antibody with strong reaction to GP_1_ was used in the subsequent western blots. As shown in Figure 2C, the GP_1_ protein exhibited faster mobility than GP_1,2_ protein by SDS-PAGE after treatment with DTT, indicating that GP_1,2_ was efficiently cleaved into GP_1_ and GP_2_ in BmN cells. And we also revealed that GP_1,2_ formed a trimeric structure in BmN cells, for three bands were detected in the native gel separation (Figure 2D), which is the appropriate size of trimer and monomer of GP. GP_1_ has several N-glycosylation sizes, so PNGase F treatment decreased the molecular weight of GP_1_ clearly, after treatment, GP_1_ migrated faster, it localized between 95 kDa and 72 kDa in both samples (Figure 2E, black arrow), which were smaller than untreated GP_1_ (Figure 2C). Expression profile indicated that GP can be detected in BmN cells from 24 to 72 h p.i., the highest yield was achieved at 48 h p.i., while the expression level in 72 h p.i. was decreased (Figure 2F, black arrow). Interestingly, polymer structure-like bands were found in sample of 24 h p.i. (Figure 2F, red arrows), and monomer band almost cannot be observed, this indicated that trimeric structure of GP was more stable in the early stage of infection. Thus, EBOV-GP_1,2_ was successfully expressed by BmBac-egfp-SP_gp64_-gp_1,2_ and BmBac-egfp-SP_gp1,2_-gp_1,2_ in BmN cells, confirming that EBOV-GP_1,2_ cannot rescue GP64 deletion in BmNPV infection.

### 3.3. The GP64 Signal Peptide Results in Ebola GP Localization to the Plasma Membrane of BmN Cells

**Figure 3:**
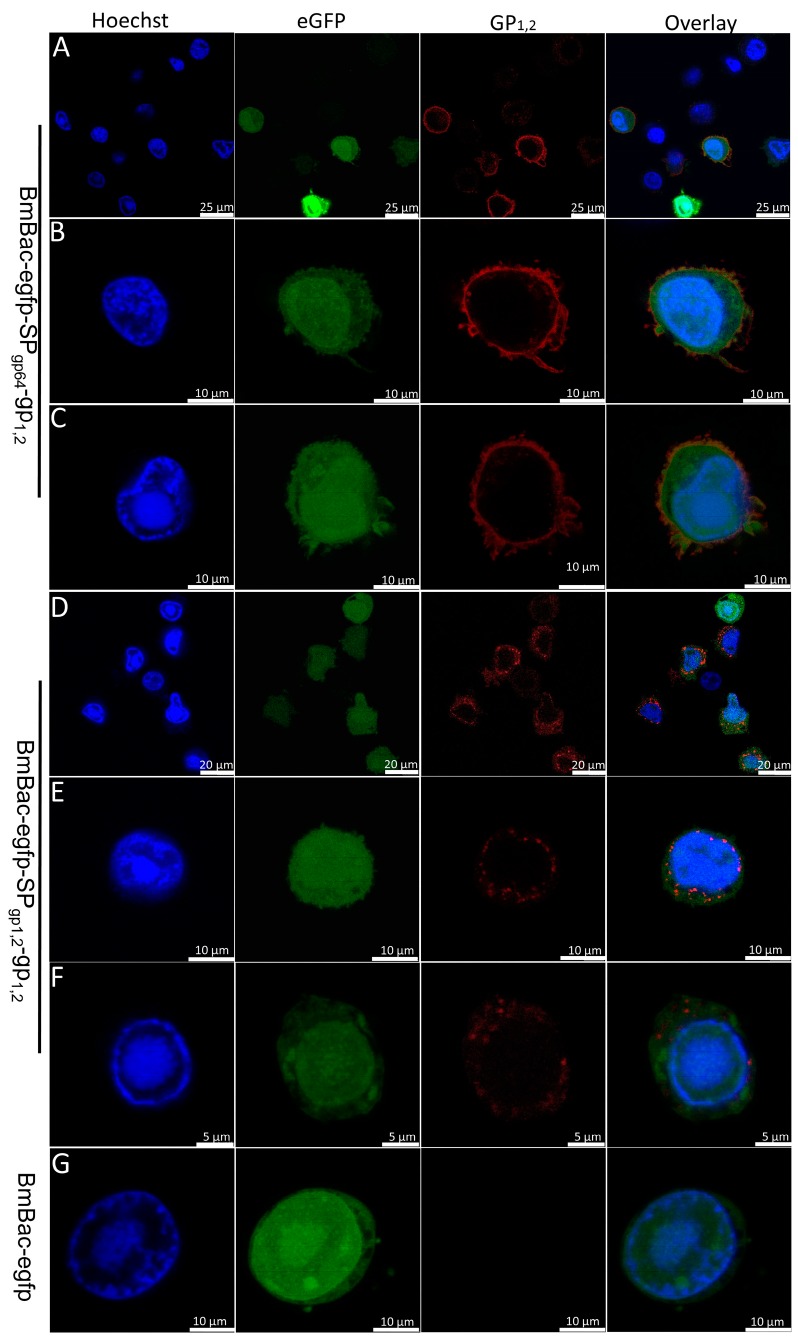
Subcellular localization of EBOV-GP in infected BmN cells. Cells were infected with BmBac-egfp-SP_gp1,2_-gp_1,2_ (**A**–**C**), BmBac-egfp-SP_gp1,2_-gp_1,2_ (**D**–**F**), and BmBac-egfp (**G**), at an multiplicity of infection (MOI) of 5 and prepared for confocal imaging at 48 h p.i. For immunofluorescence assays, monoclonal antiserum against EBOV-GP was used as the primary antibody, and Alexa Fluor 555 was used as the secondary antibody. DNA was stained with Hoechst. The panels on the right show merged images. Bar was shown in figures.

Localization of EBOV-GP in infected BmN cells was monitored by confocal microscopy. To this end, cells were grown in confocal dishes and infected with BmBac-egfp-SP_gp64_-gp_1,2_, BmBac-egfp-SP_gp1,2_-gp_1,2_, and BmBac-egfp (Figure 3A–G) and fixed, and the anti-GP antibody was applied for GP localization at 48 h p.i. Images were captured at 405 nm for Hoechst-stained DNA, 488 nm for eGFP, and 555 nm for the secondary antibody. As shown in Figure 3, green fluorescence was observed in cells infected with both viruses (Figure 3, eGFP panel), which indicates that the cells were infected efficiently. The GP protein with the GP64 signal peptide was mainly localized to the plasma membrane (Figure 3A–C, red fluorescence), and there was a filipodial shape located around the cell (Figure 3B–C). Conversely, full-length GP was uniformly distributed in the cytoplasm, no red fluorescence was observed at the plasma membrane, furthermore, GP was accumulated in cytoplasm and some red fluorescent dots were found clearly around the nucleus (Figure 3D–F). In addition, according to the green fluorescence detected, some protrusions formed around the cytoplasm. While in the cell infected with BmBac-egfp, the eGFP was distributed in the cytoplasm and nucleus, and no red fluorescence was observed (Figure 3G).

### 3.4. GP Expression Causes Protrusion Formation Around BmN Cells

**Figure 4:**
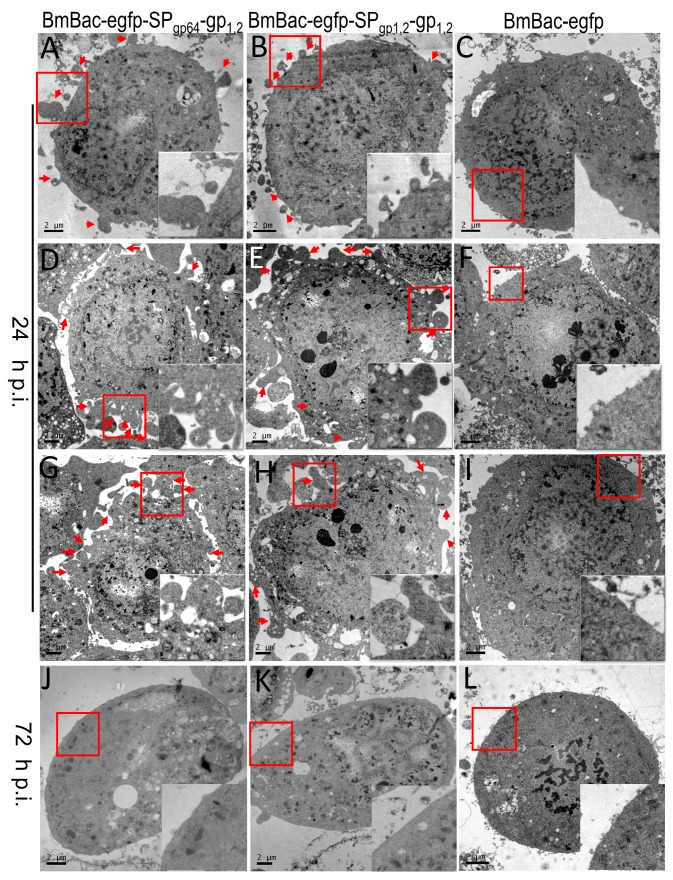
Morphological study by TEM. BmN cells infected with, BmBac-egfp-SP_gp1,2_-gp_1,2_ (**A**,**D**,**G**,**J**), BmBac-egfp-SP_gp1,2_-gp_1,2_ (**B**,**E**,**H**,**K**), and BmBac-egfp (**C**,**F**,**I**,**L**) at 24 h p.i. and 72 h p.i. Arrows show the protrusions produced by virus infection, boxed regions of the cell membrane are magnified and shown in the inset.

Cells were infected with viruses for 24 h, fixed and subjected to TEM analysis. Based on observations of the ultra-thin sections, GP-expressing cells showed a significant change in morphology (Figure 4). Many large protrusions were formed around the cell membrane (Figure 4A,B,D,E,G,H, red arrows), though almost no protrusions formed in control cells (Figure 4C,F,I). This result was consistent with the confocal observation that protrusions formed around infected cells. When cells were infected for 72 h, the cell membrane was smoother, and there were almost no protrusions (Figure 4J,K); therefore, GP expression can induce BmN cells to produce protrusions, resulting in dramatic morphological changes in the early stage of infection.

## 4. Discussion

Baculovirus has been widely used for recombinant protein production. In this study, we successfully expressed the full-length Ebola glycoprotein using the BmNPV baculovirus expression system. According to western blot results, GP can be expressed and processed into GP_1_ and GP_2,_ (Figure 2). GP_2_ reacted strongly with the monoclonal antibody, the two bands may result from insufficient glycosylation, whereas the GP_1_ reaction to monoclonal antibody was weak. In the subsequent western blots, including native gel, reducing, nonreducing gel separation and deglycosylation assay, we used polyclonal antibody for which the reaction with GP_1_ was very effective, these may be associated with the high density of O-linked glycans situated within a serine- and threonine-rich region in the C-terminal half of GP_1_ [22], our result verified that GP_1_ was N-glycosylated in BmN cells. GP expression caused strong cytotoxicity in host cells, resulting in cell damage and possibly facilitating immune escape by the virus [23], and also induced dramatic morphological changes [24]. We confirmed these morphological changes at the early stage of infection (24 h p.i.), which almost disappeared at 72 h p.i. (Figure 4). Vesicle protrusions formed around infected cells, and some vesicles were released into the medium, indicating that GP expression activates vesicle formation and release in BmN cells, similar to the situation for mammalian cells [24] and Sf9 cells [25]. Here, we showed that GP_1,2_ formed a more stable polymer structure in 24 h p.i. in reducing gel, which was not detected in 48 or 72 h p.i., this may be associated with the characteristic of polyhedrin promoter, it produced large scale protein which cannot be folded correctly in late-stage of infection. Thus, dramatic GP expression-induced morphological changes may be associated with vesicle formation and release, which is determined by the characteristics of EBOV-GP, and trimeric GP formation may play a key role but need further verification.

In addition, GP_1,2_ was cleaved into GP_1_ and GP_2_ by furin-like proteases, which are linked through an intersubunit disulfide bond and noncovalent interactions in the Golgi [3,8], we here verified that GP_1_ and GP_2_ were linked by disulfide bond by nonreducing gel separation, and GP_1,2_ form trimeric structure in BmN cells which is the mature structure of EBOV-GP in viron [26]. Moreover, GP_1,2_ with both signal peptides produced GP_1_ and GP_2_ in this study, which indicates that GP_1,2_ was translocated and secreted into the Golgi for processing. GP_1,2_ with the native peptide lacked transmembrane secretion ability in BmN cells, hereby, GP_1,2_ accumulated in the cytoplasm to form dots distribution which may result from the secretion failure. However, the BmNPV viral signal peptide efficiently compensated for this defect (Figure 3). This finding indicates that the EBOV-GP signal peptide is inefficient for GP transmembrane secretion in insect cells and that GP_1,2_ transmembrane localization requires the viral signal peptide to overcome transmembrane defectiveness in BmN cells. Accordingly, we suggest that BmN cell-BmNPV expression systems are suitable for EBOV-GP expression.

BmNPV lost infectivity when membrane fusion protein GP64 was deleted from the BmNPV genome. Although we showed that GP_1,2_ can be expressed and processed in normal viruses, when GP_1,2_ was restored in BmBac^∆gp64^, no amplification was observed in the transfected cells, and the GP64-restored virus can amplify as a control. Despite the inability to detect GP_1,2_ expression due to its lower level, we suggest that GP_1,2_ is expressed in BmN cells transfected with BmBac^∆gp64^ harboring GP_1,2_. However, EBOV-GP can replace vesicular stomatitis virus glycoprotein (VSVG), the same class III membrane fusion protein with GP64, to produce recombinant virus (V920) [27], V920 replicated well in Vero cells, while no replication was observed in relevant arthropod cells [28]. Hereby, GP_1,2_ cannot rescue GP64 deficiency in BmNPV infection, this may result from the variability of the NPC-1 receptor, which may provide information for anti-EBOV research.

In addition, EBOV-GP pseudotyped virus particles have been widely used for vaccine preparation because of their strong protection against viral infection [29], such as influenza virus [30], and HIV-1 [31], V920 [27]. V920 is currently being assessed in a phase III efficacy trial [32], which shows 100% protection against Ebola Virus Disease during the West African Ebola epidemic in 2015, and it has subsequently been deployed for emergency control of Ebola outbreaks in central Africa [33]. Nonetheless, 77.34% preexisting immunity to adenovirus in the general healthy population was reported in Guangzhou [34], as well as in South Africa and other regions with a higher prevalence [35], which is a major obstacle to the clinical application of adenovirus-based pseudotyped virus vaccines. The baculovirus expression vector system is generally regarded as among the most promising and versatile eukaryotic expression systems currently available [36]. As a safe vector, baculovirus has been approved by the FDA for vaccine preparation and cannot replicate in mammalian cells. For instance, the human papillomavirus virus-like particle vaccine (Cervarix, GlaxoSmithKline) produced by the baculovirus expression system has been commercialized for the prevention of cervical cancer. There is a long history of raising silkworms in China, and silkworms are safe for the environment and easily bred at low cost. Thus, the BmNPV expression system is suitable for GP vaccine preparation.

## 5. Conclusions

In summary, our study is the first to demonstrate that EBOV-GP can be expressed in BmN cells using the BmNPV bacmid system. Although differing signal peptides led to differential localization of GP, this variation showed no effect on protein processing, as both were cleaved into GP_1_ and GP_2_ to form trimeric GP. Nonetheless, cleaved EBOV-GP cannot substitute for the function of GP64. This study enhances our understanding of GP expression and processing in BmN cells, which may contribute to EBOV-GP expression using baculovirus. However, potential applications for vaccine preparation based on BmNPV require further investigation.

## Figures and Tables

**Table 1:** Primers used in this study.

Primers	Sequence (5′–3′, Restriction Endonuclease Site Underlined)
Upstream arm	*AACAAAAAAGCAATCTCATAACCACCATGGAGAACACCAAGTTTGGCGGC*GCACCAATAACTGCCTTAA
Downstream arm	*CTATACAATTTTTTTTATTACAAATAATGATACAATTTTTATTATTACAT*CTGTCCTTCCTGTGCGA
GP64UP	CGCGAATTCGACAGATATTTAAATAAACCAAAC
CmRP	CTGTCCTTCCTGTGCGA
PGP64-F:	GGCAGGCCTGACAGATATTTAAATAAGCCAAAC (*Stu*I)
PGP64-R	CGCTCTAGATGAGGCATCTTATATACCCGA (*Xba*I)
GP1,2 F	CGCTCTAGAATGGGCGTTACAGGAATATTG (*Xba*I)
GP1,2 no SP F	GGCTCTAGACCACTTGGAGTCATCCACAAT (*Xba*I)
GP1,2 R	CCCAAGCTTCTAAAAGACAAATTTGCATAT (*Hin*dIII)

Nucleotides homologous to the *gp64* region are in italic font.
